# Enhanced wound healing properties of guar gum/curcumin-stabilized silver nanoparticle hydrogels

**DOI:** 10.1038/s41598-021-01262-x

**Published:** 2021-11-08

**Authors:** Sakkarin Bhubhanil, Chanon Talodthaisong, Mattaka Khongkow, Katawut Namdee, Prapimpun Wongchitrat, Werayut Yingmema, James A. Hutchison, Sarawut Lapmanee, Sirinan Kulchat

**Affiliations:** 1 Pre-Clinical Department, Faculty of Medicine, Siam University, Bangkok, 10160 Thailand; 2 Department of Chemistry, Faculty of Science, Khon Kaen University, Khon Kaen, 40002 Thailand; 3 National Nanotechnology Centre (NANOTEC), National Science and Technology Development Agency, Pathumthani, 12120 Thailand; 4 Center for Research and Innovation, Faculty of Medical Technology, Mahidol University, Nakon Pathom, 73170 Thailand; 5 Laboratory Animal Center, Thammasat University, Pathumthani, 12120 Thailand; 6 School of Chemistry, The University of Melbourne, Parkville, VIC 3010 Australia

**Keywords:** Chemical biology, Chemistry

## Abstract

Biocompatible materials that act as scaffolds for regenerative medicine are of enormous interest. Hydrogel-nanoparticle composites have great potential in this regard, however evaluations of their wound healing and safety in vivo in animal studies are scarce. Here we demonstrate that a guar gum/curcumin-stabilized silver nanoparticle hydrogel composite is an injectable material with exceptional wound healing and antibacterial properties. We show that the curcumin-bound silver nanoparticles themselves exhibit low cytotoxicity and enhance proliferation, migration, and collagen production in in vitro studies of human dermal fibroblasts. We then show that the hydrogel-nanoparticle composite promotes wound healing in in vivo studies on rats, accelerating wound closure by > 40% and reducing bacterial counts by 60% compared to commercial antibacterial gels. Histopathology indicates that the hydrogel composite enhances transition from the inflammation to proliferation stage of healing, promoting the formation of fibroblasts and new blood vessels, while target gene expression studies confirm that the accelerated tissue remodeling occurs along the normal pathways. As such these hydrogel composites show great promise as wound dressing materials with high antibacterial capacity.

## Introduction

Functional bio(nano)materials research, including towards new drug delivery systems and enhanced scaffolds for regenerative medicine, is a fast-developing field of the life sciences^1,2^. Hydrogel biomaterials have been of much interest in this context as these three dimensional (3D), water absorbing and retaining, natural polymer networks have been shown to facilitate healing^3,4^. Moreover, they can be biocompatible, have shape memory^5^, be self-healing^6^, injectable^7^, soft^1^, and have a rubbery texture which is similar to human tissues. Hydrogels are key elements in numerous biotechnology/biomedical applications in the field of tissue engineering^2^, wound dressing^8^, and drug delivery^9,10^. Hydrogels based on polysaccharides^11^ have received considerable attention due to their low cost, natural abundance, biodegradability, and low toxicity. While many hydrogels cannot be injected via syringe, shear-thinning hydrogels have recently overcome this limitation, as they display viscous flow under shear stress but fast recovery of stiffness after stress removal^12^. In this work, we selected Guar Gum (GG) as the gelator to form injectable hydrogels. It is extracted from the seeds of Cyamopsis tetragonoloba and is composed of β-1,4-linked mannose residues with a branch of α-d-galactopyranose units at the 6-position^7^. GG has various applications in the biological and food industry due to its being non-toxic, more soluble than other biopolymers, inexpensive, biodegradable, and a facile gelator at room temperature^13,14^. In addition, GG is biocompatible and employed in medical contexts such as in wound dressings^15^, drug delivery^16^, and artificial tissue engineering^17^ because it has very similar properties to native body tissues and retains function *in vivo*^18^.

Wounds are physical injuries, including acute traumas or surgery, resulting in an opening or breaking of the skin. Subsequent infections present non-trivial health problems to patients, driving significant developments in wound treatment^8,13,14^. Proper healing of wounds is essential for the restoration of disrupted anatomical stability, to shorten healing time, to decrease the risk of infection, and to restore functional status to the skin^15^. Repair of injured tissues, including regeneration and replacement stages, occurs in a sequence of events consisting of inflammation, proliferation, and migration of different cell types^16,17^. The inflammation stage begins immediately after injury, first with vasoconstriction that favors homeostasis and releases inflammation mediators^18^. The most important function of this stage is not to repair damaged tissue but to stop blood flow to the wound. The proliferative phase is characterized by granulation tissue proliferation, driven mainly by fibroblasts and the angiogenesis process. At this stage, new blood vessels and fibroblasts in the extracellular matrix generate myofibroblasts to decrease the size of the wound^19^. Finally, the remodeling or maturation stage is characterized by reformulation and improvement of collagen fibers along tension lines to restore normal toughness to the skin^17,20^.

Wound healing is thus a complicated process that can be affected by several factors including infection, stress, contamination, medication, and sex hormones. Recently, many medications have become available on the world market to treat/heal wounds^21^. However, these treatments exhibit limitations based on cost, treatment time, and toxicity (side effects). Current synthetic wound healing medications protect the wound from infection but do not speed up the healing process and can have several side effects. Natural product-based treatments may exhibit several advantages including biocompatibility, effectiveness, and ease of extraction from natural sources. In this work we focus on using natural, biodegradable, and injectable hydrogels composited with wound healing agents for in vivo wound healing investigations.

Various wound healing agents, including anti-inflammatories and antioxidant free radical scavengers, have been reported^21,22^. Curcumin or *curcuma longa* is a plant commonly used as a culinary ingredient but which also exhibits potent antioxidant, antiseptic, anti-inflammatory, blood purifying, and wound-healing agent properties^23^. It also inhibits lipid peroxidation and scavenges superoxide anion, singlet oxygen, nitric oxide, and hydroxyl radicals^24–26^. However, its poor water solubility and fast degradation profile compromise its bioavailability upon administration^27^. Improved biodistribution of native curcumin has been a target for a battery of nanotherapeutics, for example by coating curcumin at the surface of nanoparticles as a delivery vector. Many nanoparticles are intrinsic therapeutic agents themselves, for example silver nanoparticles (AgNPs) show broad-spectrum antimicrobial properties against fungi and bacteria, including antibiotic-resistant strains^28^. They are now commonly used in biomedical applications for wound dressing and management^29,30^. The use of natural products such as curcumin as stabilizers during the synthesis of AgNPs is cheap and eco-friendly^31,32^, but may also enhance their therapeutic activity. However scarce reports exist of the in vivo efficacy of plant extract-stabilized AgNPs.

**Figure 1:**
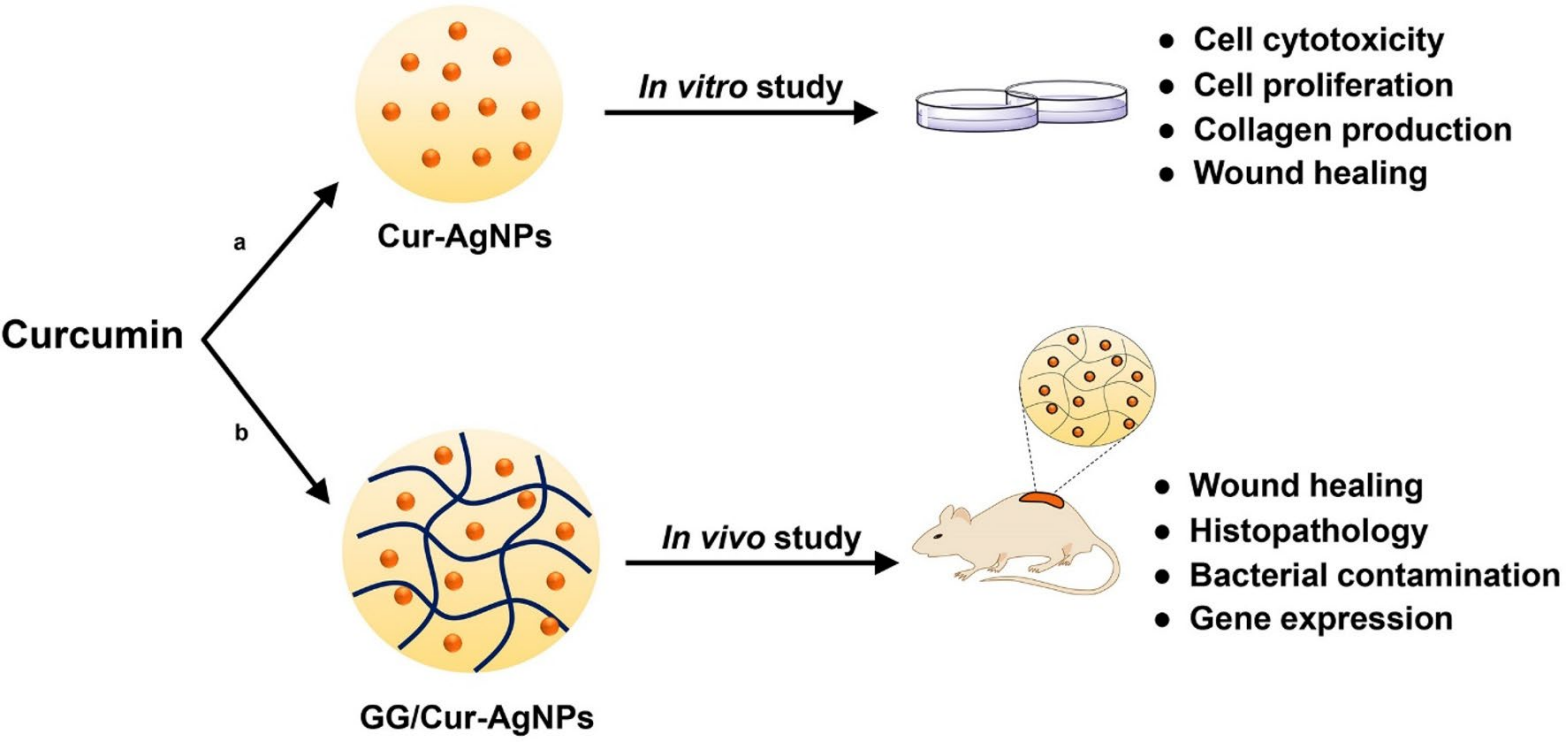
Overview of the study of enhanced wound healing using curcumin-silver nanoparticles (in vitro) and their hydrogel composites (in vivo). Curcumin is used as a surface-binding agent to stabilize silver nanoparticles (Cur-AgNPs) which are then composited into guar gum hydrogels (GG/Cur-AgNPs). (**a**) Cur-AgNPs were investigated for cell cytotoxicity, cell proliferation, collagen production, and wound healing in in vitro studies. (**b**) GG/Cur-AgNPs were studied for wound healing, histopathology, bacterial contamination, and gene expression in vivo on rats.

The current work addresses these issues. We report a biodegradable, injectable hydrogel nanoparticle composite prepared using guar gum (GG) and composited with silver nanoparticles stabilized by curcumin (GG/Cur-AgNPs)^7^. We find that the Cur-AgNPs have low cytotoxicity and enhance the proliferation and migration of, and collagen production within, human dermal fibroblasts. We then apply the composite hydrogel to full thickness incisional wounds in rats (Fig. 1), assessing histological changes, and expression of wound healing-related genes. The GG/Cur-AgNPs hydrogels enhance wound healing and antibacterial efficiency by > 40% and 60% respectively compared to commercial antibacterial gels for wound treatment.

## Results and discussion

### Synthesis and characterization of Cur-AgNPs and shear-thinning, injectable hydrogels

**Figure 2:**
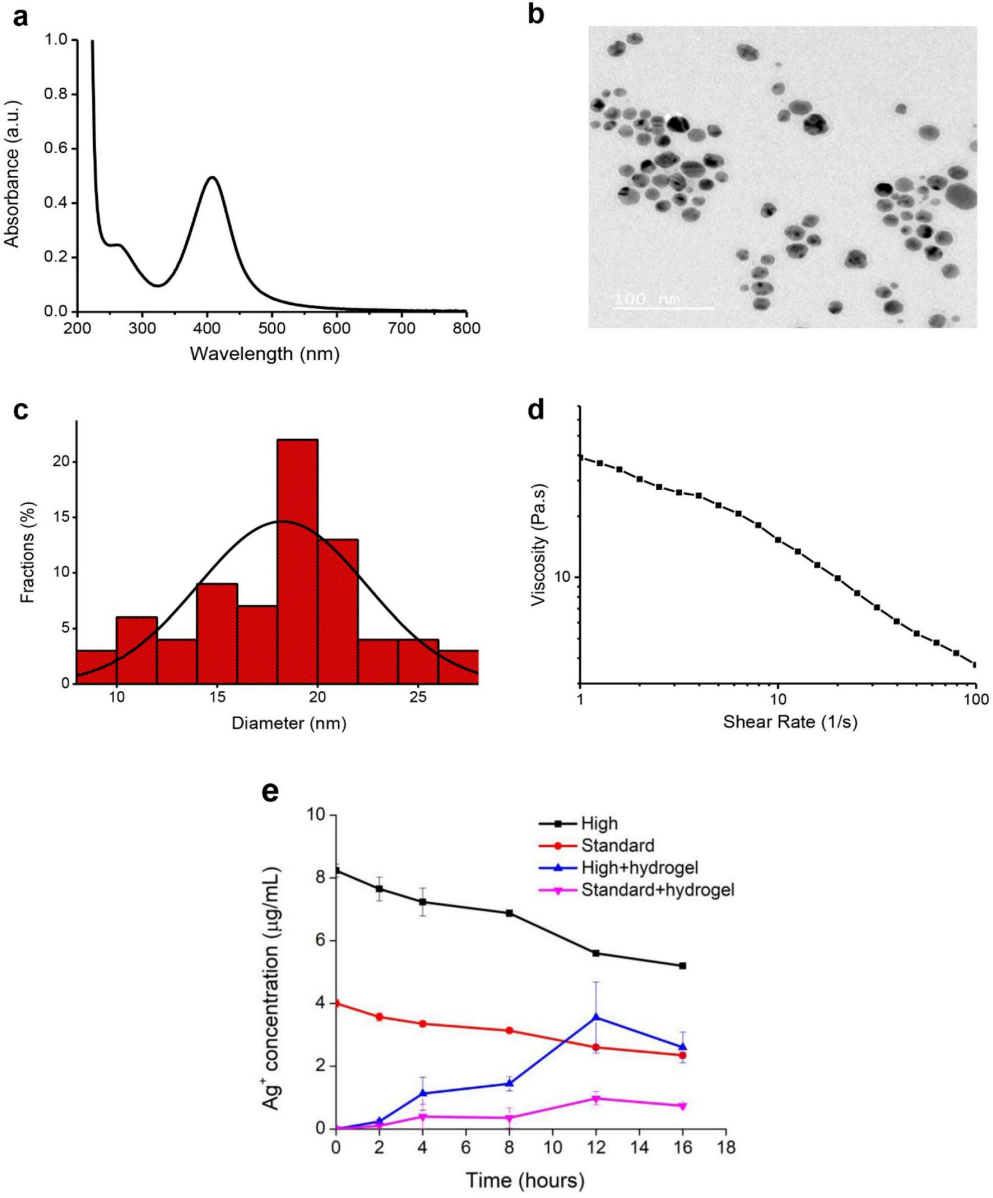
Characterization of Cur-AgNPs and shear-thinning properties of the composite GG/Cur-AgNPs self-healing hydrogel. (**a**) UV–Vis spectrum of Cur-AgNPs. (**b**) TEM image of Cur-AgNPs. (**c**) the average diameter of Cur-AgNPs is 18.24 ± 4.20 nm (n = 75) from TEM. (**d**) Viscosity of the hydrogel composite as a function of shear rate. The hydrogel shows shear thinning which is a favorable property for injectability. **e**, The concentration of silver ions (Ag^+^) released by ‘high’ (1000 μg/mL) and ‘standard’ (500 μg/mL) concentrations of Cur-AgNPs, and for the same concentrations of Cur-AgNPs encapsulated in the GG hydrogel.

Silver nanoparticles stabilized by curcumin (Cur-AgNPs) were synthesized following a previously reported method^7^ and characterized using UV–vis spectroscopy and transmission electron microscopy (TEM) as shown in Fig. 2. The extinction spectrum of aqueous solutions of Cur-AgNPs display a well-defined surface plasmon resonance band at 407 nm (Fig. 2a), which together with the electron microscopy studies suggests a homogenous dispersion (a nanoparticle diameter distribution of 18.24 ± 4.20 nm (n = 75) was determined from TEM). AgNPs in this size range have been associated with high antimicrobial activity and low cytotoxicity previously^33^. The hydrodynamic size and the zeta potential values of aqueous Cur-AgNPs were also determined, giving values of 51.9 ± 0.8 nm (PDI = 0.428) and − 25.2 ± 1.4 mV respectively. NP diameters are normally found to be larger by DLS than by TEM as the former measures the ligand/water layer around the particle, though particle aggregation in solution may account for the difference herein. The high negative surface charge however indicates that the Cur-AgNPs are well-stabilized and we observed no settling of Cur-AgNP dispersions over months^34,35^.

The hydrogel was prepared using guar gum as a gelator and borax as a cross-linker with a weight ratio of 5:1 (guar gum : borax) and then composited with the Cur-AgNPs to obtain GG/Cur-AgNPs following a previous report^7^. The GG hydrogel was characterized by ATR-FTIR spectroscopy as shown in Fig. S1 showing characteristic peaks at 3328 cm^−1^, 2897 cm^−1^, 1642 cm^−1^, and 1017 cm^−1^ indicating the presence of OH stretching, C-H stretching, C-H bending, and C–O–C stretching vibrations, respectively. The morphology of the GG hydrogel (Fig. S2) indicates spaces/pores between scaly and fractured materials. The thermogravimetric analysis results as shown in Fig. S3 reveal initial moisture loss in the region of 25–250 °C followed in the zone 250–300 °C by guar gum backbone degradation. All these results fully match a previous report^7^. As observed previously, the GG/Cur-AgNPs composite exhibits fast self-healing and excellent elastomeric behavior^7^.

Here we performed additional rheological characterization showing that the hydrogel composite (GG/Cur-AgNPs) is suitable for injection through a syringe, a favourable property for biomedical applications. We performed shear rate sweep measurements at different shear rates in the range of 1–100 s^−1^, confirming shear-thinning behavior in the composite GG/Cur-AgNPs hydrogel. As shown in Fig. 2d, the hydrogel viscosity decreases as a function of the shear rate, indicating that the hydrogel exhibits fluid-like flow under shear force, a critical property for easy injection through a syringe^36^. The hydrogel recovers a gel state when the shear force disappears.

As the release of silver ions from AgNPs has been associated with toxicity, the release of silver ions (Ag^+^) from Cur-AgNPs and from GG-Cur-AgNPs was determined using inductively coupled plasma optical emission spectrometry (ICP-OES) (Fig. 2e). The results show that silver ions are released very gradually from Cur-AgNPs, taking many hours and in proportion to the concentration of Cur-AgNPs present. Silver ion release is however much slower from the GG/Cur-AgNPs hydrogel composites, suggesting the latter are safer to use on wounds.

### The effect of Cur-AgNPs on cell cytotoxicity, cell proliferation and collagen production in human dermal fibroblast cells

**Figure 3:**
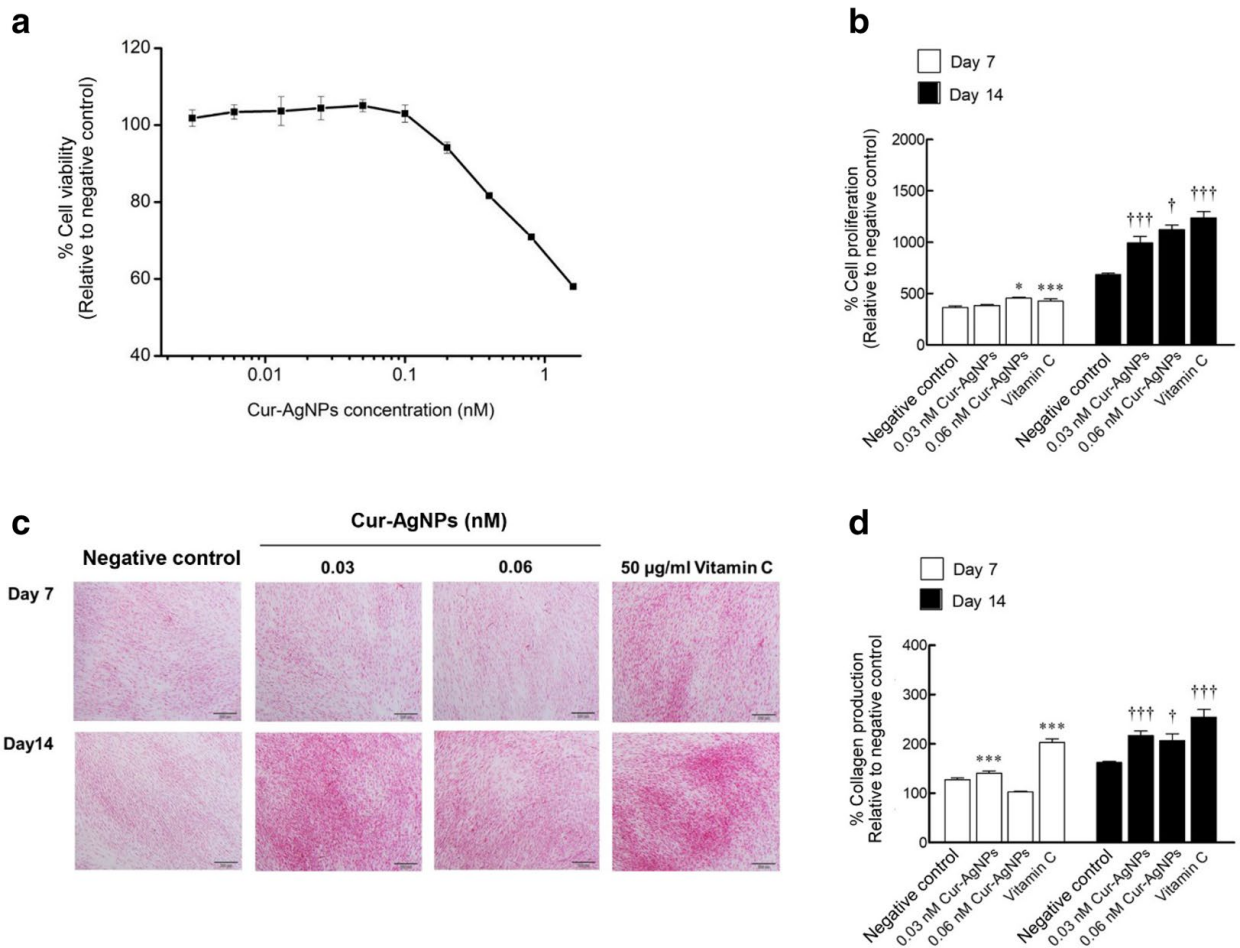
Effects of Cur-AgNPs on cell viability, cell proliferation, and collagen production. (**a**) Human dermal fibroblast cells were treated with 0.0016 − 1.60 nM of Cur-AgNPs for 24 h in cytotoxicity testing and in the absence of Cur-AgNPs as a control. (**b**) Cell proliferation; cells were treated with 0.03 and 0.06 nM of Cur-AgNP and 50 µg/mL of ascorbic acid (Vitamin C) for 7 and 14 days in the measurement of cell production. (**c, d**) White light transmission microscopy images of human dermal fibroblast cells treated with 0.03 and 0.06 nM of Cur-AgNPs and 50 µg/mL of ascorbic acid (Vitamin C) for 7 and 14 days. Collagens are stained red by Picrosirius dye and photographed at 200 × magnification. (**e**) Analyzed percentage of collagen production relative to controls*.* Data were expressed as mean ± SD (*n* = 3). *P < 0.05 and ***P < 0.001 compared to control within 7 days post-treatment. ^†^*P* < 0.005 and ^†††^*P* < 0.001 compared to control within 14 days post-treatment.

Cell viability investigations were undertaken to ensure that the Cur-AgNPs composited in our hydrogels are not themselves cytotoxic. An MTT assay was undertaken using human dermal fibroblast cells in the presence of Cur-AgNPs at different concentrations in the range of 0.016–1.600 nM and after incubation for 24 h. As demonstrated in Fig. 3a, Cur-AgNPs have low cytotoxicity with more than 80% of the cells remaining viable after the addition of 0.200 nM Cur-AgNPs. Below 0.100 nM Cur-AgNPs, 100% relative viability was maintained. Thus, Cur-AgNPs can be considered non-cytotoxic and biocompatible at or below a concentration of 0.200 nM.

In addition, we observed enhanced proliferation and collagen production inside the fibroblasts in the presence of Cur-AgNPs. The cells were treated for 7 and 14 days with 0.03 nM and 0.06 nM of Cur-AgNPs, respectively. Ascorbic acid (50 µg/mL) was used as a positive control as it is a known stimulatory agent of both dermal fibroblast proliferation and collagen production. Cell viabilities were measured using a CellTiter-Glo Luminescent cell viability assay kit. As shown in Fig. 3b, the number of cells present increased in Cur-AgNPs-treated samples in a dose- and time-dependent manner. After 7 days, cell proliferation in the sample treated with 0.06 nM Cur-AgNPs even exceeded that of the ascorbic acid positive control. After 14 days, cell proliferation increased by 31% and 45% for 0.03 nM and 0.06 nM Cur-AgNPs relative to negative controls (absence of Cur-AgNPs), respectively.

Total collagen in the samples was visualized using Picrosirius red staining as shown in Fig. 3c–d. The cells incubated with 0.03 nM of Cur-AgNPs showed an increase in collagen of 20% after 7 days compared to the negative control (no Cur-AgNPs), though for 0.06 nM Cur-AgNPs a reduction of 20% was observed. Nevertheless, after 14 days, collagen production increased by 50% and 40% in the presence of 0.03 nM and 0.06 nM of Cur-AgNPs respectively relative to the negative controls.

In vitro experiments have shown that both the size and surface coating of AgNPs effects their activity in cultures of animal and human cells^37–39^. Here, human dermal fibroblast cells exposed to Cur-AgNPs increased proliferation and collagen production compared to untreated controls. The Cur-AgNPs also showed low cytotoxicity. These results stand alongside previous work suggesting that AgNPs mediate stimulation of transcriptional changes towards improved skin appearance^40,41^. This reflects the function of fibroblasts in mediating the secretion of extracellular matrix facilitating collagen production which corresponds to the re-epithelialization phase of wound healing^42^. Wong et al. however observed that citrate-coated silver nanoparticles of 10 nm mean diameter reduced collagen production levels in fibroblast cells in mice^43^. Further work is required to determine to what extent the curcumin surface coating and the size of the AgNPs employed herein contribute to the favourable in vitro properties of Cur-AgNPs.

### The effect of Cur-AgNPs on in vitro wound healing

**Figure 4:**
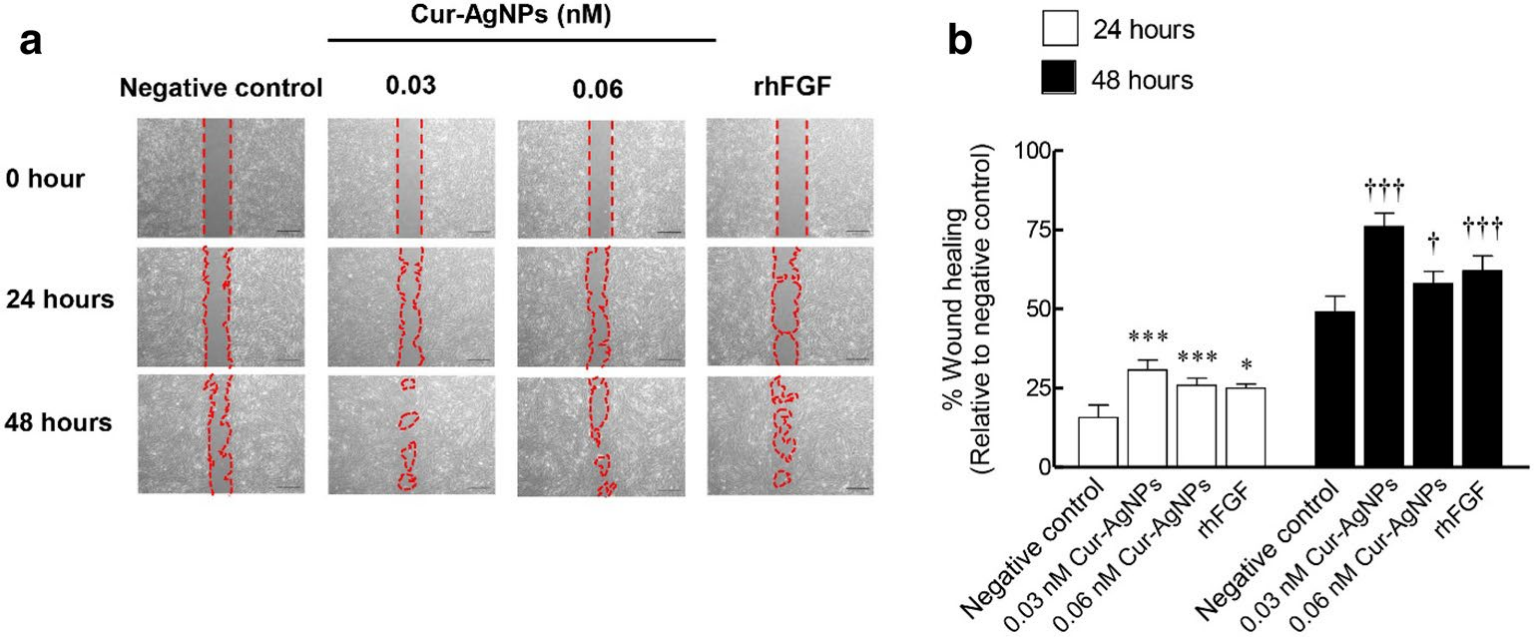
Effects of Cur-AgNPs on wound healing induction. (**a**) Human dermal fibroblast cells were treated with 0.03 and 0.06 nM of Cur-AgNP, or 100 ng/mL of rhFGF-b as a positive control, and a scratch wound assay performed at 0, 24 and 48 h after scratch wounding (compared to no treatment as a negative control). (**b**) Analyzed percentage wound gap width after 48 h relative to the original scratch width. Data were expressed as mean ± SEM (*n* = 3). *P < 0.05 and ***P < 0.001 compared to control within 24 h post-treatment. ^†^*P* < 0.005 and ^†††^*P* < 0.001 compared to negative control within 48 h post-treatment.

To examine whether Cur-AgNPs have a stimulatory effect on cell migration, a scratch wound-healing assay was performed. Human dermal fibroblast cells were treated with 0.03 and 0.06 nM of Cur-AgNPs, and recombinant human basic fibroblast growth factor (rhFGF-b) at a concentration of 100 ng/mL was used as a positive control. As shown in Fig. 4a,b, Cur-AgNPs significantly decreased the wound gap in a time-dependent manner. Treatment with 0.03 nM of Cur-AgNPs gave the best performance, showing 100% and 50% increased cell migration at 7 and 14 days relative to the negative control, and even 15–20% enhancement compared to the rhFGF-b positive control. The better performance for the lower concentration of Cur-AgNPs may be because wound healing experiments are necessarily performed with injured cells exposed to a free gap, resulting in a lack of intercellular junctions and communication. Without these key factors, most adhesive cells would be considerably weakened and unhealthy^44^. Therefore, treatment with higher concentrations of Cur-AgNPs (0.06 nM) might exacerbate toxicity to these injured cells compared to lower doses (0.03 nM). Thus Cur-AgNPs could promote wound healing by facilitating the proliferation and migration of fibroblasts to initiate granulation tissue formation/remodeling within the wound^45^.

### Enhanced wound healing and anti-bacterial effects of composite GG/Cur-AgNPs hydrogel on rat incisional wounds

**Figure 5:**
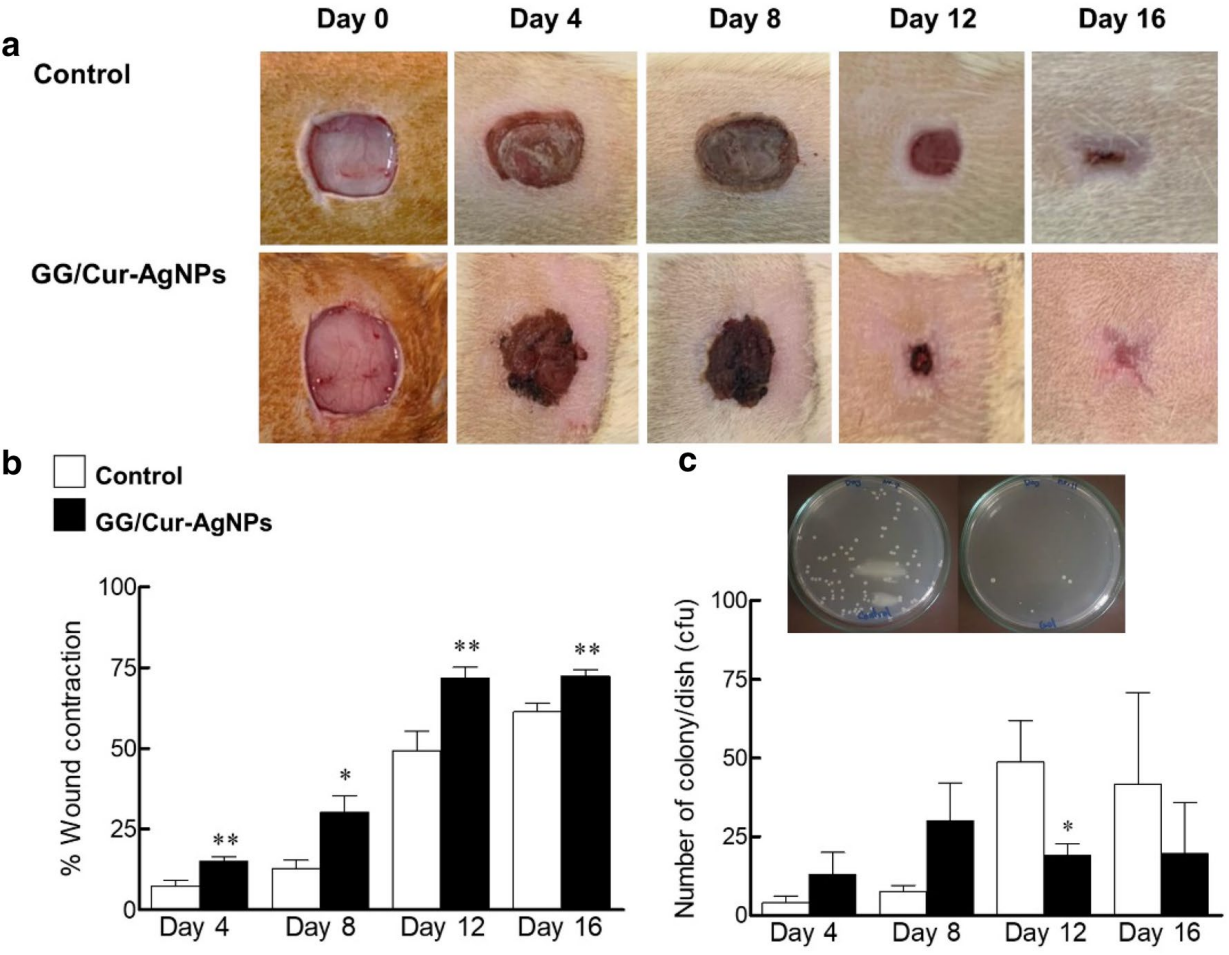
Effects of GG/Cur-AgNPs on wound healing and bacterial contamination in rats. (**a**) Time-dependent evolution of rat skin wound closure observed for treatment with commercial antibacterial gels (control) and with the GG/Cur-AgNPs hydrogel composite. (**b**) Percentage wound area contraction calculated on days 4, 8, 12, and 16 post-wound incision for treatment with commercial antibacterial gel and GG/Cur-AgNPs hydrogel composite. **c,** Comparison of bacterial colony count (CFU) at the wound site for treatment with commercial antibacterial gel and the GG/Cur-AgNPs hydrogel composite. Data were expressed as mean ± SEM (*n* = 3–4/time point). *P < 0.05 and **P < 0.01 compared to control on day of experiments.

To determine the effects of GG/Cur-AgNPs hydrogel composites on in vivo incisional wounds, GG/Cur-AgNPs hydrogels were applied to surgical wound incisions on rats and compared with commercial antibacterial gels, guar gum (GG), and Cur-AgNPs as controls. The surgical wounds treated with the GG/Cur-AgNPs hydrogel showed increased wound contraction compared to commercial antibacterial gel controls (Fig. 5a,b). The percentage wound area contraction in rats treated with guar gum and Cur-AgNPs alone are shown in Fig. S4. At 12 days post-incision, the percentage wound contraction was highest for the GG/Cur-AgNPs hydrogel (73% versus 51% for the commercial antibacterial gel, 27% for the Cur-AgNPs alone, and 19% for the guar gum hydrogel alone). The GG/Cur-AgNPs thus induced > 40% faster wound contraction than the commercial antibacterial gel.

The antibacterial effect of the hydrogel composite at the wound site was also compared to that of commercial antibacterial gel (Fig. 5c), and guar gum hydrogel and Cur-AgNPs alone (Fig. S4), via a colony count (CFU) at the wound. The GG/Cur-AgNPs hydrogel showed the lowest bacterial count on day 12 post-incision (20 cfu versus 51 cfu for the commercial antibacterial gel, 51 cfu for the guar gum hydrogel alone, and 25 cfu for the Cur-AgNPs alone). This represents a 60% higher antibacterial activity compared to commercial antibacterial gels.

### Histopathology of rat skin wound healing with GG/Cur-AgNPs hydrogel treatment

In the initial inflammatory stage of healing, typically for 3–5 days post-injury, active neutrophil infiltration is followed by monocyte recruitment into the wound, the latter differentiating into macrophages which drive transition to the proliferative phase^46,47^. The latter is characterized by granulation tissue proliferation, driven mainly by fibroblasts and angiogenesis. The populations of various cells and structures at the wound thus reveals information on healing progression, with monocytes (mononuclear leukocytes) appearing round with kidney-shaped nuclei, neutrophils (polymorphonuclear leukocytes) having lobed nuclei and cytoplasmic granules, fibroblasts having spindle shapes, and blood vessel formation indicated by capillary structures.

**Figure 6:**
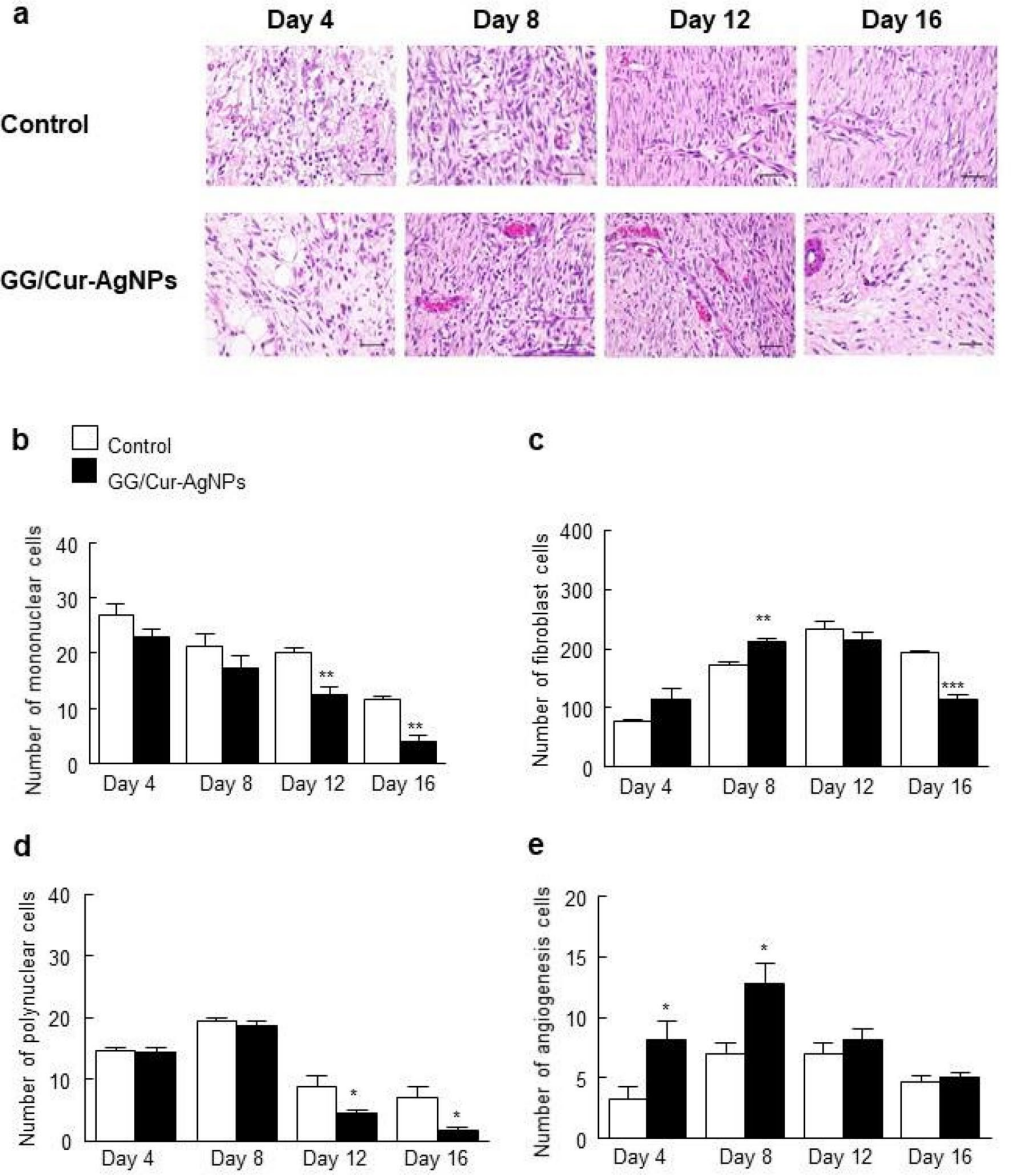
Histopathology of rat skin on day 4, 8, 12 and 16 of the wound incisions stained with hematoxylin & eosin staining. (**a**) Micrographs of sections of wound incision rat skin under treatment with commercial antibacterial gels (control) and GG/Cur-AgNPs. Hematoxylin (deep blue-purple) stains nucleic acids/nuclei, eosin (pink) stains proteins non-specifically indicating the cytoplasm and extracellular matrix. (**b**) Number of mononuclear leukocytes. (**c**) polymorphonuclear leukocytes. (**d**) fibroblasts. (**e**) capillaries were counted in 10 high-power field (HPF) (400 × magnification). Data were expressed as mean ± SEM (*n* = 3/time point). *P < 0.05, **P < 0.01 and *P < 0.001 compared to the control on day of experiments. Scale bar = 40 μm.

**Figure 7:**
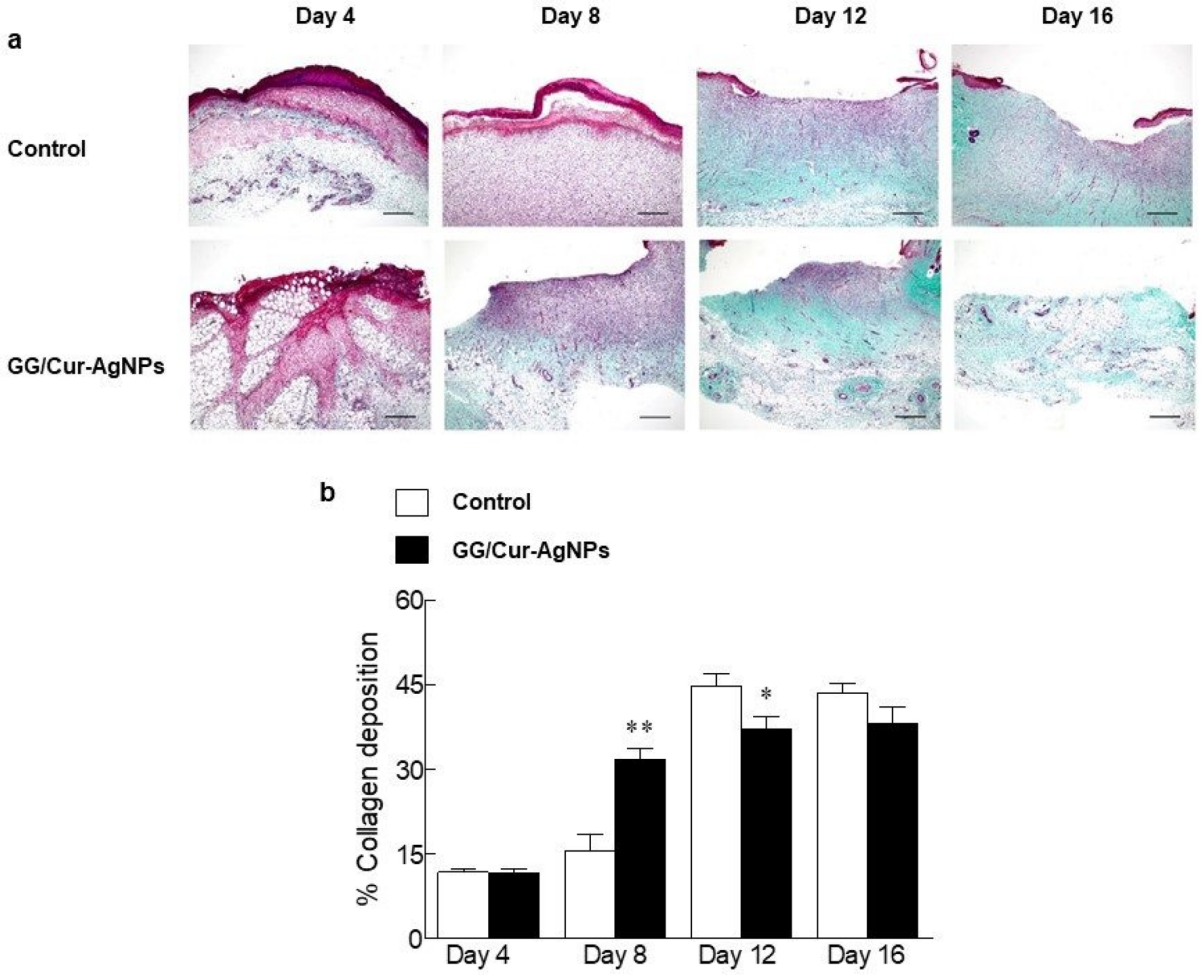
Histopathology of rat skin wounds on day 4, 8, 12 and 16 of the wound incisions stained with Masson's trichrome. (**a**) Micrographs of sections of wound incision rat skin treated with commercial antibacterial gel (top) and with GG/Cur-AgNPs (bottom). Collagen is indicated by the blue-green colour. (**b**) The percentage of collagen deposition (area of blue-green colour) was measured in 10 high-power field (HPF) (400 × magnification). Data were expressed as mean ± SEM (*n* = 3/time point). *P < 0.05 and **P < 0.01 compared to the control on the day of experiments. Scale bar = 40 μm.

Micrographs from rat skin wounds show clear differences in cell populations for treatment with GG/Cur-AgNPs hydrogel and a commercial antibacterial gel control (Fig. 6a). Monocytes and polynuclear leukocytes are strongly reduced in the wound area at 12 and 16 days post-treatment with GG/Cur-AgNPs compared to a commercial antibacterial gel, indicating the inflammatory phase finished faster (Fig. 6b,d). Meanwhile fibroblasts and capillaries are greatly enhanced on day 4 and day 8 relative to the commercial gel, indicating the proliferative phase also commenced faster (Fig. 6c,e). Re-epithelialization is necessary for complete wound healing, creating epithelial cell migration^48^. It was calculated using the following formula^49^: length of newly formed epidermis layer/length of wound between wound edges × 100. Although complete wound covering did not occur until day 16, the percentage re-epithelialization significantly increased in the GG/Cur-AgNPs group on day 8, 12, and 16 (Figs. S5a and S5b). Furthermore, GG/Cur-AgNPs increased collagen deposition, important in all stages of wound healing, at day 4 and 8 (Fig. 7a,b) compared to the commercial antibacterial gel control. These results suggest that the enhanced wound-healing effects of GG/Cur-AgNPs hydrogels involves fast transition to, and promotion of, the proliferative phase via formation of new blood vessels and neovasculation^50^.

### Changes in target genes promoting wound healing in rat incisional skin wounds treated by GG/Cur-AgNPs hydrogels

**Figure 8:**
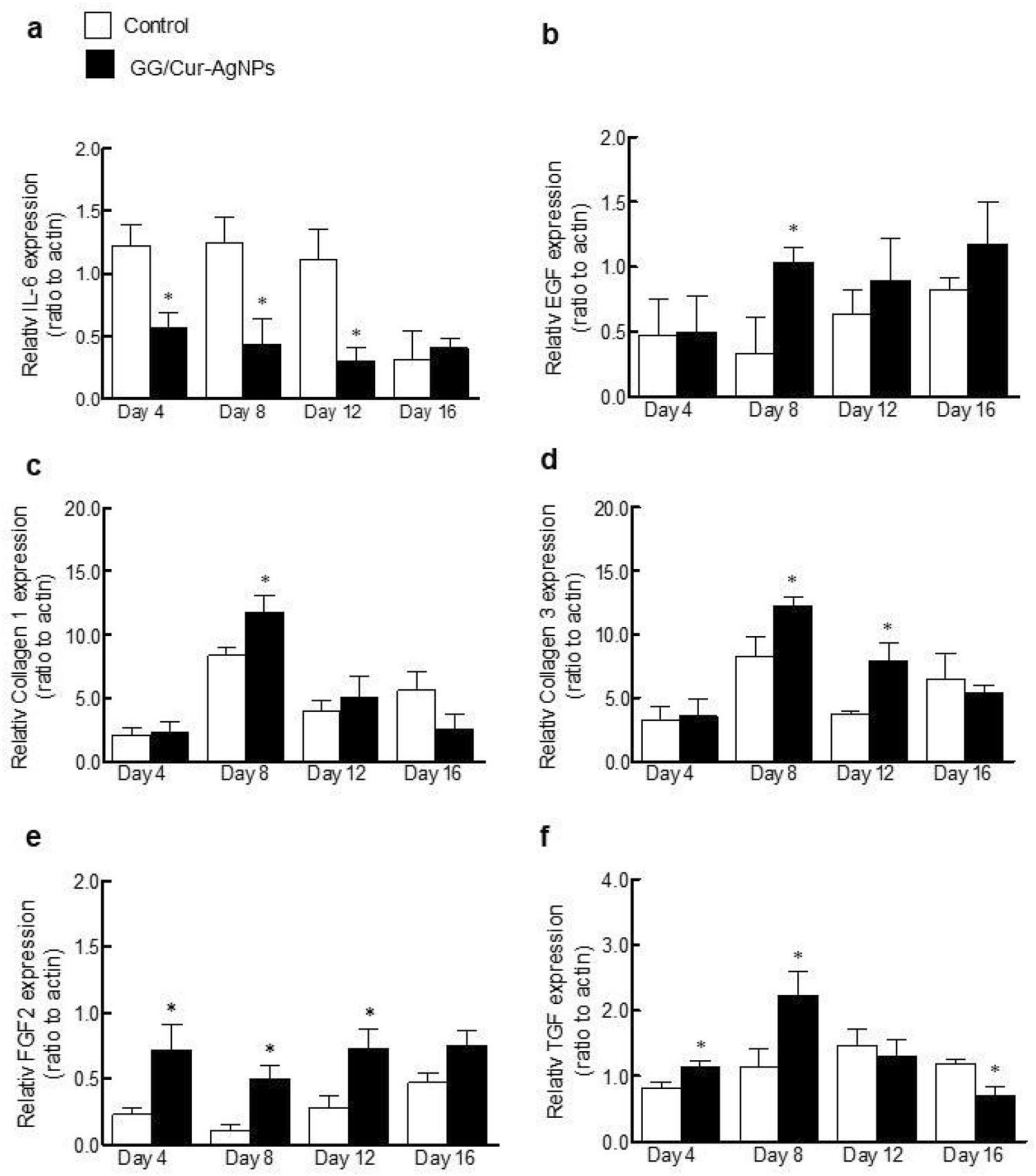
Changes of wound related genes in the healed wound skin incision with GG/Cur-AgNPs hydrogel treatment. (**a**) Relative gene expression levels of IL-6. (**b**) EGF. (**c**) collagen 1. (**d**) collagen 3. (**e**) FGF2. (**f**) TGF-β*1* were measured by RT-qPCR analysis. Data were expressed as mean ± SEM (*n* = 3/time point). *P < 0.05 compared to a commercial antibacterial gel control on the day of experiments.

Healing response involves several pathways at the molecular level that combine to modulate inflammation and promote wound closure. To determine the effects of GG/Cur-AgNPs hydrogels on the expression of target genes promoting wound healing in rat incisional skin wounds, the mRNA expressions of interleukin 6 (IL-6), epidermal growth factor (EGF), collagen-1, collagen-3, fibroblast growth factor (FGF2) and transforming growth factor β1 (TGF-β1) were investigated by RT-qPCR, relative to a commercial antibacterial gels (Fig. 8)^44^.

The results showed a decrease in the expression of IL-6 in GG/Cur-AgNPs hydrogel treatment after 4, 8 and 12 days relative to the control, but no difference in expression was observed after 16 days (Fig. 8a). The decrease of IL-6 expression may be associated with the modulation of keratinocyte differentiation and reduction of the inflammation rate^51^.

The expressions of EGF, collagen-1, and collagen-3 were increased for GG/Cur-AgNPs hydrogel treatment after day 8 (Fig. 8b–d) relative to the control. In addition, GG/Cur-AgNPs hydrogels upregulated the expression of collagen-3 gene 12 days after treatments (Fig. 8d). Furthermore, the relative expression of FGF2 was increased for GG/Cur-AgNPs hydrogel treatment after 4, 8 and 12 days (Fig. 8e) relative to the control. The expression of TGF-β1 was increased after 8 days in GG/Cur-AgNPs hydrogel-treated rats compared with controls, but after 14 days, GG/Cur-AgNPs hydrogel treatment showed a decrease in TGF-β1 expression (Fig. 8f). The observed upregulation at earlier times post-incision of these important genes involved in the inflammatory reaction, cell growth, and collagen formation, further confirms the positive effect of GG/Cur-AgNPs on wound healing. Specifically, the expression of TGF-*β*1 has a crucial role in initiating the inflammatory response in early stages of wound healing, protecting the affected area from bacterial infection. Later, secretion of EGF and FGF2 growth factors help to stimulate the proliferation phase by inducing epithelial cell migration and proliferation within the wound area. Finally, the newly synthesized collagen, mediated by collagen-1 and collagen-3 expression, promotes tissue remodeling and subsequent scar formation following wound closure^52^. These results confirm that GG/Cur-AgNPs promotes wound healing by accelerating the normal healing pathways.

### Outlook

We have shown that the GG/Cur-AgNPs composite is a shear-thinning, thus injectable, hydrogel that when applied to in vivo rat skin wounds exhibits superior wound healing and antibacterial action compared to commercial gels (by > 40% and 60%, respectively). The enhanced performance is due to accelerated transition from the inflammatory to proliferative phase of healing, with stimulation of the latter. This compares favorably with related studies. For example, Tavakoli *et al*^53^ describes a nanocomposite hydrogel based on polydopamine-modified zinc oxide nanoparticles (ZnO/PD) in Kappa carrageenan KaMA matrix with the addition of L-glutamic acid for treating diabetic wounds. This nanocomposite hydrogel showed a 10% enhancement of wound closure compared to controls. Meanwhile, Jiang *et al*^54^ showed that a self-healing hydrogel based on chitosan and konjac glucomannan (KGM) and incorporating AgNPs to improve skin tissue healing by 20% compared to controls.

We note firstly that all these advantages are offered by a hydrogel composite prepared by a facile method and with cheap, and majority natural, starting products. The shortened inflammation stage observed may be significant as prolonged inflammation can cause incomplete healing and induce scar formation. Furthermore, wound dressing investigations focused on traditional antibiotics are reaching a critical juncture, with the USA Centers for Disease Control predicting increasing deaths from antibiotic-resistant bacteria^55^. Wound dressing materials that do not depend on traditional antibiotics will thus assume increasing importance. Hydrogels composited with metallic nanoparticles may be a suitable alternative material for biomedical research and for incorporation into wound care treatment products in the marketplace.

## Materials and methods

### Materials

Silver nitrate (AgNO_3_, 99.9%) was purchased from POCH™, Poland. Curcumin synthetic grade (C_21_H_20_O_6_, pure > 97%) was purchased from TCI, Japan. Dimethylsulfoxide (DMSO, C_2_H_6_OS) was purchased from Fisher Scientific, UK. Potassium carbonate (K_2_CO_3_, ≥ 99.0%) was purchased from Merck, Germany. Guar gum was purchased from Chemipan Corporation Co., Ltd, Thailand. Sodium Hydroxide (NaOH, 99%) was purchased from RCL Labscan. Di-sodium tetraborate decahydrate) (Borax, Na_2_[B_4_O_5_(OH)_4_]8H_2_O, 99.5%) was purchased from QRëC, New Zealand. Deionized water (DI) with specific resistivity of 18.2 MΩcm was obtained from a RiOs™Type I Simplicity 185 (Millipore water purification system). Direct red 80 (C_45_H_26_N_10_Na_6_O_21_S_6_), L-ascorbic acid (C_6_H_8_O_6_, 99%), picric acid (C_6_H_3_N_3_O_7_, ≥ 98%), paraformaldehyde (HO(CH_2_O)_n_H), and agar powder were purchased from Sigma-Aldrich (St. Louis, MO, USA). Ethanol absolute (CH_3_CH_2_OH, 99.5%), hydrochloric acid (HCl, 37%), sodium hydroxide (NaOH, 99.0%) were purchased from Carlo Erba Reagents, Val de Reuil, France. 3-4,5-Dimethylthiazol-2-yl)-2,5-diphenyltetrazolium bromide (C_18_H_16_BrN_5_S, 98%) was purchased from Merck Millipore Calbiochem (Massachusetts, USA). Recombinant human FGF-b (rhFGF-b) was purchased from American Type Culture Collection (ATCC, Manassas, Virginia, USA. Dulbecco’s modified Eagle’s media (DMEM), fetal bovine serum (FBS), penicillin and streptomycin (100 µg/mL) were purchased from Gibco, USA. CellTiter-Glo Luminescent cell viability assay kit and lysis buffer reagent were purchased from Promega Corporation, Wisconsin, USA. Micro-dishes were obtained from ibidi GmbH, Gräfelfing, Germany. Regarding animal studies, antibacterial gels as a control were purchased from Union Drug Laboratories Ltd. Bangkok, Thailand. Hematoxylin and eosin Trichrome reagents were purchased from Sigma-Aldrich, St. Louis, MO, USA. For the gene expression studies, DNase I and SuperScript II kit were purchased Thermo Fisher Scientific Inc, USA. The primers were designed from Geneplus Co Ltd, Huai Khwang, Bangkok.

### Preparation of guar gum-curcumin stabilized silver nanoparticle hydrogel (GG/Cur-AgNPs)

Silver nanoparticles stabilized by curcumin (Cur-AgNPs) were synthesized following our previous publication^7^. Briefly, a solution of 20 mM curcumin in DMSO (750 μl) was added to 68 mL of DI water in a 100 mL round bottom flask. The solution pH was then adjusted to 10 by 0.07 M K_2_CO_3_ and the solution heated up to 95 °C. Next, 7.5 mL of 10 mM AgNO_3_ was quickly added to the solution mixture. The mixture was stirred vigorously at 100 °C for 1 h and filtered by micro filter to obtain the Cur-AgNPs. The concentration of Cur-AgNPs was estimated to be 3.14 nM based on an extinction coefficient of 41.8 × 10^8^ M^−1^ cm^−1^ at 400.8 nm for 20 nm diameter citrate-silver nanoparticles^56^. The GG/Cur-AgNPs hydrogel was prepared by a modification of our previous publication^7^. Briefly, 0.1 g of guar gum was dissolved in 20 mL of the Cur-AgNPs solution. Then, 0.1 M NaOH (200 μL) and 4 wt% borax (500 μL) were added to the solution. The mixture was then stirred until gelation occurred to obtain yellow hydrogels designated GG/Cur-AgNPs.

### Transmission electron microscopy (TEM) analysis

TEM observations were made using an Atomic Resolution Analytical Electron Microscope (JEM-ARM200F; JEOL) at an acceleration voltage of 200 kV. Cur-AgNPs were freshly prepared on a TEM grid (Ultra-Thin PELCO Grids for TEM; Ted Pella).

### Dynamic light scattering and zeta potential measurement

Hydrodynamic diameters and zeta potential of Cur-AgNPs were investigated by dynamic light scattering (DLS) and electrophoretic light scattering, using a Malvern Zetasizer Nano series (Nano ZS, UK). The samples measurements were performed in triplicate and the data represented as mean ± standard deviation (SD).

### Rheological study of shear thinning hydrogel

The shear thinning behavior of GG/Cur-AgNPs hydrogel was examined using a parallel-plate (smooth stainless steel, 25 mm diameter) rheometer (Physica MCR500, Germany). The viscosity and shear rate were investigated under rotation mode at 25 ℃ with shear rate from 1 to 100 s^−1^ and frequency was kept constant at 1 rad⋅s^−1^.

### Silver ion (Ag^+^) release investigation

The concentration of silver ions (Ag^+^) released from Cur-AgNPs and the hydrogel composite was monitored using inductively coupled plasma optical emission spectrometry (ICP-OES). Solution concentrations of Cur-AgNPs of 1000 μg/mL and 500 μg/mL were prepared. The hydrogels were prepared as discussed above with concentrations of Cur-AgNPs of 1000 μg/mL and 500 μg/mL, respectively. Then, the two hydrogels were immersed in two test tubes in the presence of 10 mL of weakly acidic solution (HNO_3_, pH = 5) at room temperature. Two different concentrations of Cur-AgNPs solutions (1000 μg/mL and 500 μg/mL) were also tested. After 2 h, 4 h, 8 h, 12 h, 16 h, 1 mL solution from test tube was placed into a small vial. Then, 1 mL of weakly acidic solution was added. Finally, all samples were monitored by ICP-OES for their silver ion concentration.

### Cell culture preparation

Human dermal fibroblast cells were purchased from ATCC (Manassas, VA, USA). Cells were cultivated in Dulbecco’s modified Eagle’s media (DMEM) (Gibco, UK) supplemented with 10% heat-inactivated fetal bovine serum (FBS), penicillin (100 U/mL) and streptomycin (100 µg/mL) (Gibco, USA). Cells were incubated at 37 °C in a humidified atmosphere containing 5% CO_2_.

### Cell viability assay

The cytotoxicity was measured by MTT assay, which determined the mitochondrial-dependent reduction of MTT [3-(4, 5-Dimethylthiazol-2-yl)-2, 5-diphenyltetra- zolium bromide Merck Millipore Calbiochem (Massachusetts, USA) to formazan. Cells were seeded at a density of 2 × 10^5^ cells/mL in 96 well-plates and incubated for 24 h. After incubation, cells were treated with various concentrations of sample for 24 h. The medium was replaced with 1 mg/mL of MTT and further incubated for 4 h at 37 °C. Then the MTT was removed and the formazan produced was dissolved with DMSO. The absorbance was measured at 570 nm using a microplate reader (Synergy H1, BioTeK).

### Cell proliferation and collagen content assay

Cell proliferation was measured by CellTiter-Glo Luminescent cell viability assay kit (Promega, USA), which evaluated the cellular ATP levels. Cells were seeded at a density of 1 × 10^5^ cells/mL in 48 well-plates and incubated for 24 h. After incubation, cells were treated with samples for 7 and 14 days. Then, the medium was removed and cells were lysed with 1X lysis buffer reagent (Promega, USA). After 10 min incubation, cell lysates were further incubated with CellTiter-Glo reagent for 10 min. The luminescence was measured using a microplate luminometer (SpectraMax L, Molecular Devices, California, USA). Collagen content was determined by Picrosirius red staining, which stained collagen type I and type III. Cells were seeded at density of 1 × 10^5^ cells/mL in 48 well-plates and cultivated for 24 h. After incubation, cells were treated with samples for 7 and 14 days. After treatment, cells were washed with PBS and fixed with 4% paraformaldehyde (Sigma-Aldrich, St. Louis, MO, USA) for 10 min. Cells were then washed twice with PBS and stained with 0.1% direct red 80 (Sigma-Aldrich, St. Louis, MO, USA) in saturated picric acid (Sigma-Aldrich, St. Louis, MO, USA) for 10 min. After staining, 0.01 N HCl in 70% ethanol was added into each well for washing excess dye. Stained collagen was visualized under an inverted microscope (CKX41, Olympus, Japan) and dissolved with 0.5 N NaOH. The amount of collagen was quantified by measuring the absorbance at 540 nm using a microplate reader.

### In vitro wound healing assay

Wound healing assays were performed to observe directional cell migration. Cell suspensions of 3 × 10^5^ cells/mL were added to each chamber of a culture-insert 4 well in µ-dish 35 mm (ibidi GmbH, Gräfelfing, Germany) and cultivated for 24 h. After incubation, the culture-insert was removed, and cells were washed with PBS. For each treatment type the scratch was applied and the wound area photographed at 0, 24 and 48 h to monitor cell migration. The filling of the gap in the scratch area, i.e. wound closure, was examined by measuring changes in wound area using imageJ. The percentage gap-filling can be calculated by the following Eq. (1).

1 $$\%\text{ gap filled }= \frac{{\mathrm{A}}_{t=0h }-{A}_{t=nh}}{{\mathrm{A}}_{t=0h }} \times100$$

A_t=0h_ is the wound area measured immediately after wounding; A_t=nh_ is the wound area measured after wounding at each time point.

### Animals and surgery of skin incision

Sixteen male Wistar rats (8-week-old and 180–200 g weight) were purchased from Nomura Siam International Co., Ltd., Bangkok, Thailand. Rats were housed in groups (2 rats/cage) under controlled condition (12 h light/dark cycle, 21 °C and 50% relative humidity). Laboratory standard chow food and distilled water were provided ad libitum for the animal. This study was approved in according to ethics committee guidelines and all protocols of animal experiments by the Institution's Animal Care Committee, Thammasat University, Thailand (Protocol number 021/2562). Rats were inhaled isoflurane anesthesia and two parallel 1 cm full thickness skin incisions were made at the midline of vertebral spine^57^. All rats were regularly observed for infection. If there were signs of infection, rats were separated and excluded from the study. Wounds were cleaned daily and then GG/Cur-AgNPs hydrogels or standard antibacterial gels were applied. The skin wounds were then photographed on days 4, 8, 12, and 16 post-wounding surgery. According to Murthy et al. work^58^, percentage of wound contraction was calculated using Eq. (2):

2 $$\% \text{wound contraction}=\frac{\text{Healed wound area}}{\text{Total wound area}}\times 100$$

$$\text{Healed wound area}=\text{ original wound area }-\text{ present wound area}$$

### Histopathology

The cross-sectional full-thickness skins and deep granulation tissues were collected on days 4, 8, 12, 16 post-wounding incision for histopathological studies. Specimens were fixed in 4% buffered paraformaldehyde, dehydrated through a graded series of ethanol, cleared with xylene solutions and blocked with paraffin, respectively. Thereafter, the paraffin blocks were sectioned into 5 μm sections by a Leica microtome (Microsystems, Wetzlar, Germany) and stained with hematoxylin and eosin or Masson’s Trichrome (Sigma-Aldrich, St. Louis, MO, USA). Interpretation of histological slides were performed as a blind analysis by two pathobiologists. Three parallel sections were taken from each specimen and the parameters of cellular infiltration including the number of mononuclear leukocytes, polymorphonuclear leukocytes and fibroblasts were measured. Vascularization and collagen deposition were also qualitatively evaluated. Morphological evaluations were photographed by a Nikon DXM 1200 digital camera (Tokyo, Japan) followed by scoring of the percentage of green colored area of the granulation tissue using ImageJ analysis software.

### Microbiological examinations

Swabs were taken from the incisional wound during each dressing change on days 4, 8, 12 and 16 post-wounding surgery. The collected swabs were diluted by tenfold serial dilutions of normal saline for quantitative analysis. Six hundred microliters of each sample dilution were spread onto agar plates (1.5% agar powder, Sigma-Aldrich, St. Louis, MO, USA) and incubated at 37 °C for 24 h. Thereafter, the bacterial colony cell numbers were counted.

### Quantitative real-time PCR (qPCR)

The RNA from skin from the healing wound area was collected and extracted using TRIzol reagent (Invitrogen Life Technologies, USA) according to the manufacturer’s protocol. The RNA was treated with DNase I (Thermo Fisher Scientific Inc, USA) to eliminate contamination with genomic DNA. The cDNA synthesis was made by reverse transcription using the High Capacity cDNA Reverse Transcriptase Kit (Applied Biosystems, USA) following manufacturer’s instructions and analyzed the expression of wound healing related genes (i.e., *Col1a1*, *Col3a1*, *Fgf2*, *Tgfb1*, *Egf*, and *Il6*) using SYBR Green-based qPCR. The primers have been validated for specificity and efficiency by conventional qPCR, as previously described^44^. The details of the primers used in this study are presented in Table 1. The detail of procedures used for qPCR amplification and analysis were described in our previous reports^59–61^. Briefly, the diluted cDNA and primers were added in the SsoAdvanced™ Universal SYBR Green Supermix (Bio-Rad Laboratories, Hercules, CA, USA) for PCR amplification in total 20 µl reaction volume. PCR reactions were performed in duplicate including sample and nontemplate control reactions and were run in the CFX96 Touch™ Real-Time PCR Detection System (Bio-Rad Laboratories). The thermocycling process consisted of 40 cycles followed by an additional step for dissociation curve generation. Beta-actin (*Actb*) was included as the reference gene for normalization of the target genes and for compensation of inter-PCR variation between each qPCR experiment. In each independent experiment, target and reference gene cDNA were derived from similar extracted RNA and run simultaneously in the qPCR analysis. The relative mRNA expression was achieved with the CFX Manager™ software (Bio-Rad Laboratories, Hercules, CA, USA) by performing the comparative C_t_ method. The expression level of each studied gene is presented as fold change by relative compared to the level in untreated control group.

**Table 1:** Primers used in qPCR.

Gene	Primer sequence	Product (bp)	Melting temperature (°C)	Access number
β-actin (*Actb*)	F: 5′-CCCTGGCTCCTAGCACCAT-3′ R: 5′-GATAGAGCCACCAATCCACACA-3′	80	60	NM031144
Collagen 1 (*Col1a1*)	F: 5′-CATGTTCAGCTTTGTGGACCT-3′ R: 5′-GCAGCTGACTTCAGGGATGT-3′	94	60	NM053304
Collagen 3 (*Col3a1*)	F: 5′-GGGATCCAATGAGGGAGAAT-3′ R: 5′-CCTTGCGTGTTTGATATT-3′	128	60	NM032085
EGF *(Egf)*	F: 5′-CTCAGGCCTCTGACTCCGAA-3′ R: 5′-ATGCCGACGAGTCTGAGTTG-3′	93	60	NM012842
FGF-2 (*Fgf2*)	F: 5′-GATCCCAAGCGGCTCTACTG-3′ R: 5′-TAGTTTGACGTGTGGGTCGC-3′	105	60	NM019305
TGF-β1 (*Tfgb1*)	F: 5′-GGGCTACCATGCCAACTTCTG-3′ R: 5′-GAGGGCAAGGACCTTGCTGTA-3′	82	60	NM021578
IL-6 (*Il6*)	F: 5′AACCTGAACCTTCCAAAGATGG-3′ R: 5′-TCTGGCTTGTTCCTCACTACT-3′	168	55	NM012589

*EGF* epidermal growth factor; *TGF-β1* transforming growth factor beta 1; *IL-6* Interleukin-6; *F* forward; *R* reverse.

### Statistical analysis

Statistical analysis was performed using GraphPad Prism 6.0 (GraphPad Software, Inc., San Diego, CA). The results were expressed as mean ± standard error of the mean (SEM). The comparisons between groups were conducted using student’s-t test and/or between and within groups using one-analysis of variance (ANOVA) with post-hoc Tukey's Honest test. All statistical tests were set at a significance level α of 0.05 (*P* < 0.05).

### Ethics statement

For the animal studies, all experimental protocols were approved by the Institution's Animal Care Committee, Thammasat University, Thailand (Protocol number 021/2562). All methods were carried out in accordance with the Guide for the Care and Use of Laboratory Animals of the National Research Council. This study was carried out in accordance with ARRIVE guidelines (https://arriveguidelines.org).

## Supplementary Information

Supplementary Information.
