# Supplementary material for: Enhanced wound healing properties of guar gum/curcumin-stabilized silver nanoparticle hydrogels
Source: Sci Rep. 2021 Nov 8;11:21836. doi: 10.1038/s41598-021-01262-x (PMC8576043; doi:10.1038/s41598-021-01262-x)
Supplement: Supplementary file 1 — Supplementary Information. [file 41598_2021_1262_MOESM1_ESM.pdf]

Enhanced wound healing properties of guar gum/curcumin-stabilized silver nanoparticle hydrogels

Sakkarin Bhubhanil[1], Chanon Talodthaisong[2], Mattaka Khongkow[3], Katawut Namdee[3], Prapimpun Wongchitrat[4], Werayut Yingmema[5], James A. Hutchison[6], Sarawut Lapmanee[1]*, Sirinan Kulchat[2]*,

[1]Pre-clinical Department, Faculty of Medicine, Siam University, Bangkok, 10160, Thailand.
[2]Department of Chemistry, Faculty of Science, Khon Kaen University, Khon Kaen, 40002, Thailand.
[3]National Nanotechnology Centre (NANOTEC), National Science and Technology Development Agency, Pathumthani, 12120, Thailand.
[4]Center for Research and Innovation, Faculty of Medical Technology, Mahidol University, Nakon Pathom, 73170, Thailand.
[5]Laboratory Animal Center, Thammasat University, Pratumthani, Thailand.
[6]School of Chemistry, The University of Melbourne, Parkville, Victoria 3010, Australia.

Type of article: Research article

***To whom correspondence should be addressed:**
1. Sirinan Kulchat, Ph.D.
Materials Chemistry Research Center, Department of Chemistry and Center of Excellence for Innovation in Chemistry, Faculty of Science, Khon Kaen University, Khon Kaen, 40002, Thailand.
E-mail: sirikul@kku.ac.th
2. Sarawut Lapmanee, Ph.D.
Pre-clinical Department, Faculty of Medicine, Siam University, Bangkok, 10160, Thailand.
E-mail: sarawut.lap@siam.edu

Keywords: curcumin; hydrogel; natural gum; wound healing, antibacterial

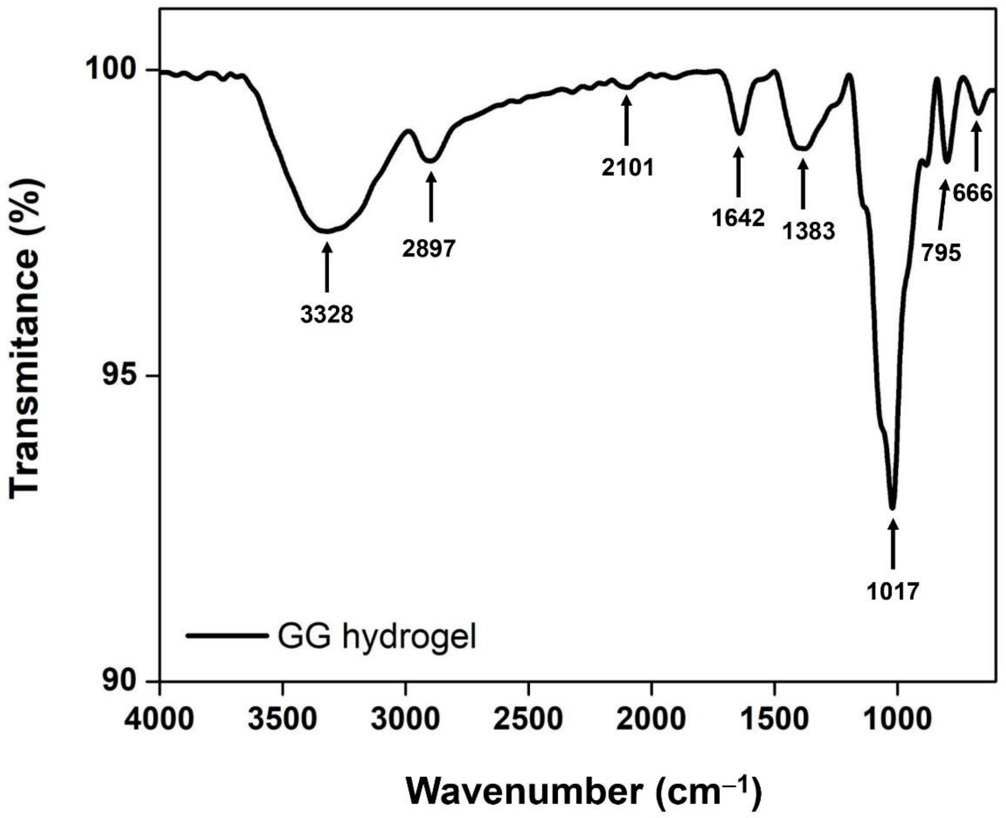

Figure S1 | ATR-FTIR spectrum of GG hydrogel

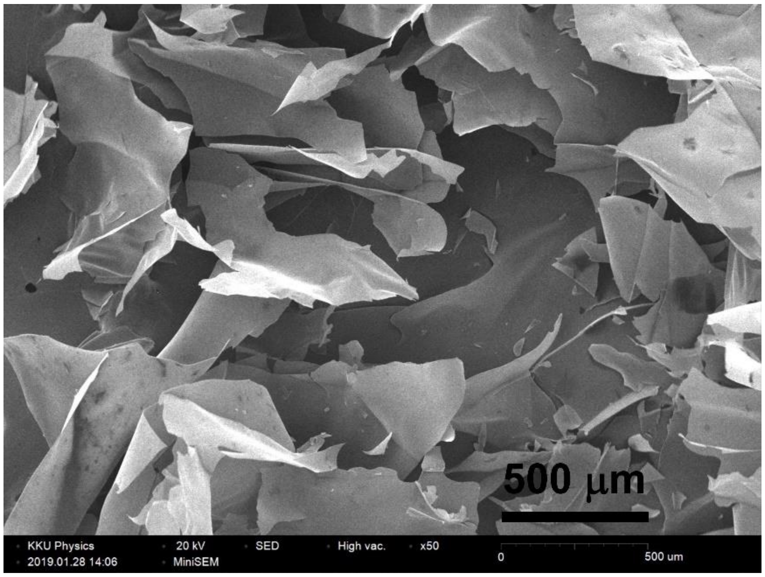

Figure S2 | Morphological studies: SEM Analysis of GG hydrogel

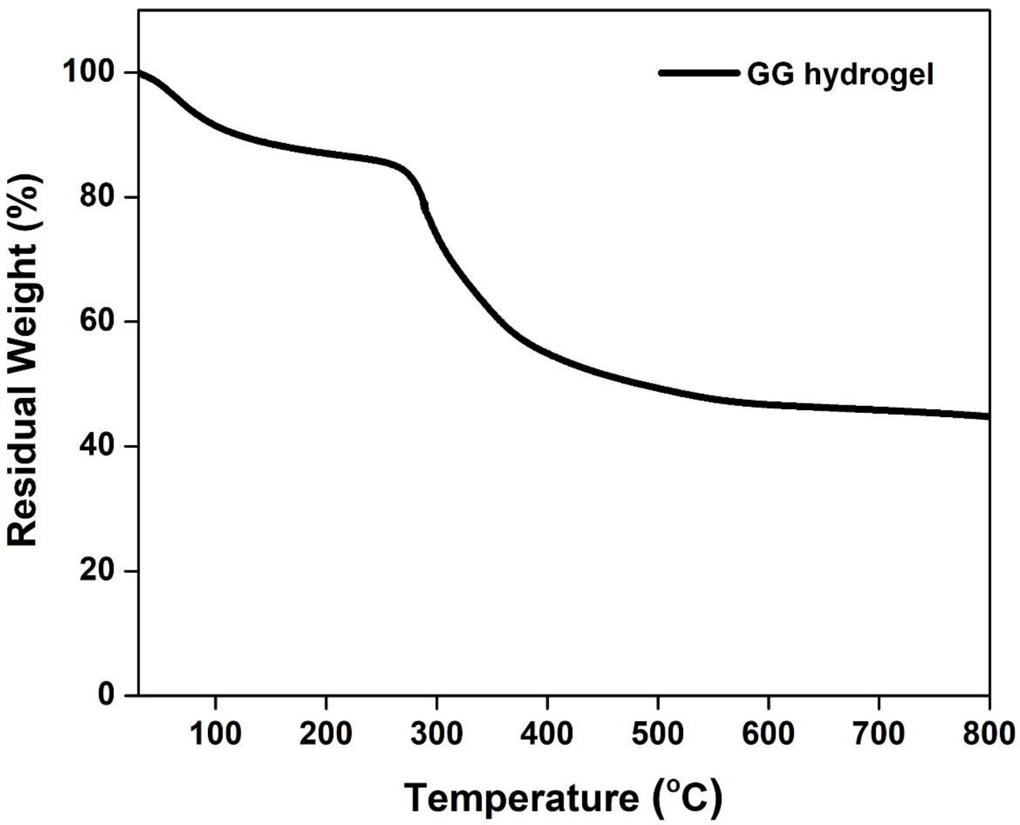

Figure S3 | Thermo gravimetric analysis of GG hydrogel.

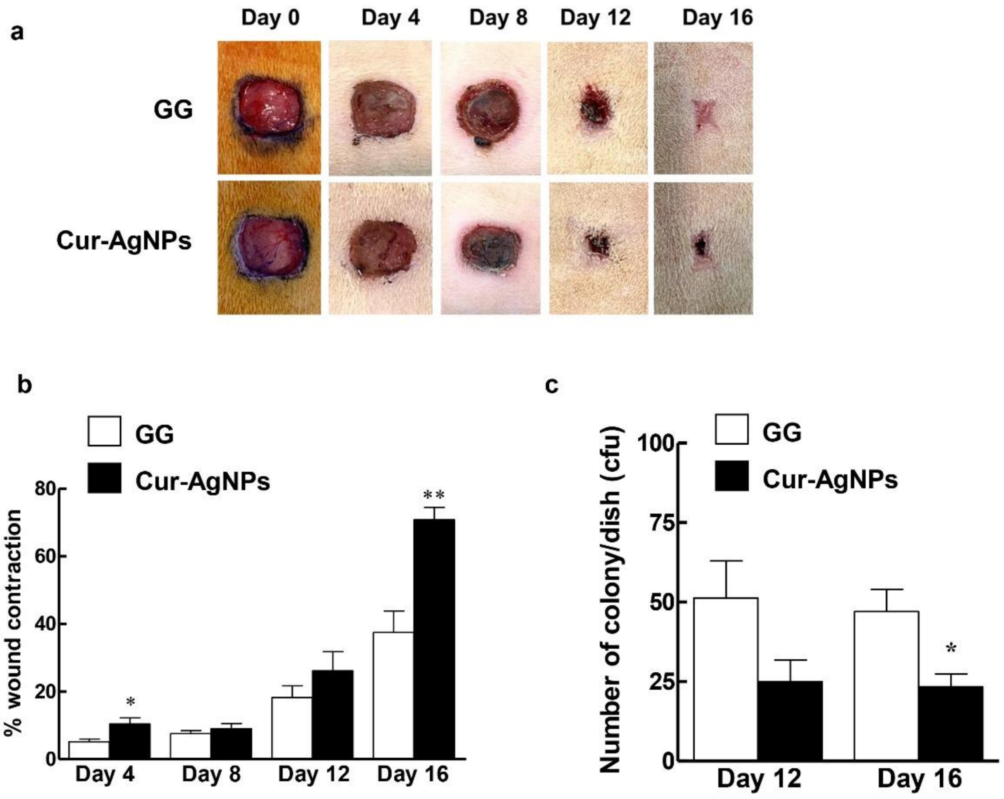

Figure S4 | Effects of GG and Cur-AgNPs on wound healing and bacterial contamination in rats. a, Time-dependent evolution of rat skin wound closure was observed for treatment with guar gum hydrogel (GG) alone, and for curcumin-stabilized silver nanoparticles (Cur-AgNPs) alone. **b,** The percentage wound area contraction was calculated on days 4, 8, 12, and 16 post-wound incision for GG and Cur-AgNPs treatment. **c,** Comparison of colony count (CFU) at the wound site for GG and Cur-AgNPs treatments. Data were expressed as mean ± SD (n = 3-4/time point). *$P < 0.05$ and **$P < 0.01$ compared to control on day of experiments.

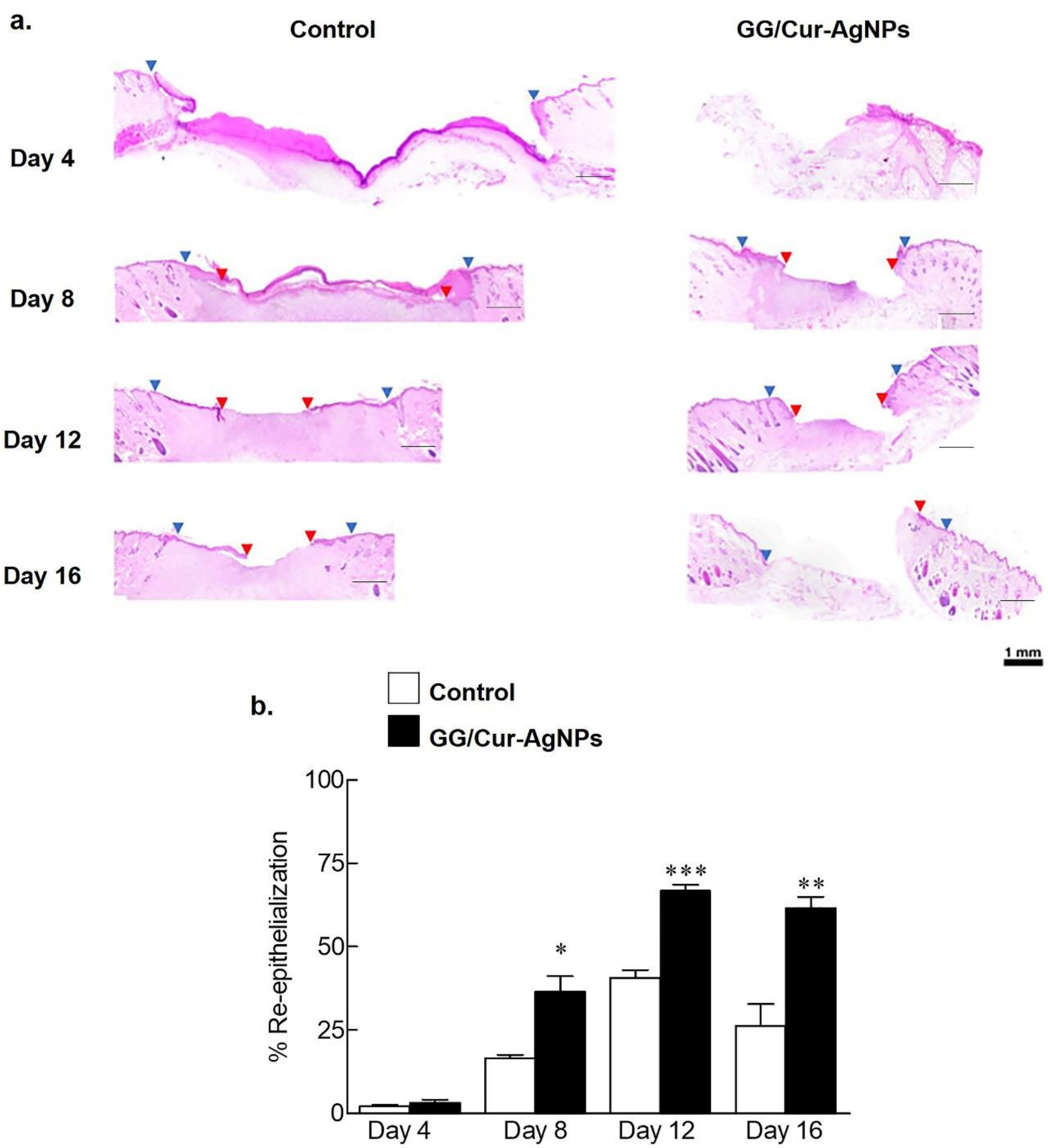

Figure S5. Histopathology of re-epithelialization (%) in rat skin on day 4, 8, 12 and 16 of the wound incisions stained with hematoxylin & eosin staining. a. Micrographs of sections of wound incision rat skin under treatment with commercial antibacterial gels (control) and GG/Cur-AgNPs. Blue arrow indicates original wound edges and red arrow indicate edge of newly formed epidermis. b, Ratio of re-epithelialization (%) were calculated [re-epithelialization length/wound length) x 100)] in 10 high-power field (HPF) (40x magnification). Data were expressed as mean ± SD (n = 3/time point). *$P < 0.05$, **$P < 0.01$ and *$P < 0.001$ compared to the control on day of experiments. Scale bar = 1 mm.
